# Long-Term Outcomes of Ventral Hernia Repair: An 11-Year Follow-Up

**DOI:** 10.7759/cureus.9523

**Published:** 2020-08-02

**Authors:** Nikita Kadakia, Ross Mudgway, Jonathan Vo, Vinson Vong, Tiffany Seto, Pascal Bortz, Aron Depew

**Affiliations:** 1 Surgery, University of California Riverside School of Medicine, Riverside, USA; 2 General Surgery, Loma Linda University School of Medicine, Loma Linda, USA; 3 General Surgery, Riverside University Health System Medical Center, Riverside, USA; 4 Emergency Medicine, University of California Riverside School of Medicine, Riverside, USA; 5 Oncology, Kaiser Permanente, San Francisco, USA; 6 Surgery, Beaver Medical Group, Redlands, USA

**Keywords:** abdomen ventral hernia, ventral wall hernias, hernia mesh

## Abstract

Background: Ventral hernia repair (VHR) is one of the most common general surgery procedures; however, few studies with long-term follow-up of VHR outcomes exist.

Methods: We performed a retrospective review of VHRs performed from 2000 to 2009 at a single institution. Our primary outcome was recurrence, and secondary outcomes were reoperations and complications including seroma, hematomas, abdominal wall abscess, wound infections, and mesh infections.

Results: Our sample population (n=420; mean age 46.3±11.7 years) included 230 females (54.8%), and cases included laparoscopic (n=31; 7.5%), laparoscopic converted to open (n=7; 1.7%), and open (n=373, 90%). As compared to suture repairs, mesh repair was associated with lower rates of complications (25.7% vs 29.5%, p=0.10) and recurrence (12.8% vs 15.2%, p=0.67). Laparoscopic repairs had lower rates of complications than open repairs (25% vs 26.8%; p=0.70) but similar rates of recurrence (13.8% and 13.6%; p=0.53). After logistic regression, obesity, chronic obstructive pulmonary disease, component separation technique, and prolonged operating time (>75th percentile) were associated with increased complications.

Conclusion: Obesity is a modifiable risk factor and must be addressed in patients undergoing VHRs. Mesh repair does not increase the risk of adverse long-term outcomes and may be performed safely in patients undergoing VHR.

## Introduction

Ventral hernia repair (VHR) is one of the most common general surgery procedures in the United States. More than 400,000 VHRs are performed annually [1]. These surgeries can be performed through open or laparoscopic techniques and with or without mesh. 

Compared to the laparoscopic approach of VHR, open surgery has been associated with similar outcomes in mortality but mildly increased morbidity [2,3]. Additionally, mesh use has been associated with a lower risk for recurrence and a higher risk of infections [4,5]. Thus, mesh use is increasingly common in VHRs. Various factors such as mesh position and mesh placement technique may affect these complication rates. Intraperitoneal is the preferred mesh position in laparoscopic repairs; however, retrorectus placement of mesh may result in a lower recurrence rate and infections [6,7]. Certain comorbidities such as obesity, smoking history, and diabetes mellitus have also been associated with increased complications [8,9]. Preexisting hypertension is associated with increased rates of complications in abdominal surgical procedures overall; however, there is a lack of evidence on hypertension as a risk factor in hernia repairs, specifically [10].

Despite the abundant literature on outcomes of VHRs, few studies have a long-term follow-up. As a result, the long-term outcomes of VHRs, specifically recurrence and complications, are not well described. There is a need to identify major predictors of long-term complications, recurrences, and reoperations in patients undergoing VHRs to guide clinical practice. Such evidence may be used to guide specific practice approaches such as body mass index (BMI) cutoffs for surgery or the optimal duration for preoperative smoking cessation. These may also be useful to general surgeons in selecting the best method for surgical repair of ventral hernias. We now report factors associated with long-term outcomes, including recurrence, complications, and reoperation in patients undergoing VHRs.

## Materials and methods

Patient selection

After institutional review board (IRB) approval, a retrospective review of paper charts and electronic medical records of all patients over age 18 years, who underwent ventral incisional hernia repair at a single institution between January 1, 2000 and December 31, 2009, was performed. Only patients undergoing repair of primary hernias or incisional hernias with no more than one recurrence were included. Suprapubic and infraumbilical hernia repairs were excluded.

Data collection

Perioperative data collected included age, sex, BMI, type of hernia, size of defect, mesh position, mesh fixation technique, comorbidities (including hypertension, chronic obstructive pulmonary disease [COPD], previous abdominal surgery), and tobacco use. Postoperative data collected included postoperative urinary retention, length of hospital stay, and operative time. Prolonged operating time was defined as operating time greater than 75th percentile (155 minutes).

Independent variables

Information about the size and type of repair was retrospectively collected. Laparoscopic repairs that were converted to open were classified as open repairs. The hernia defect size was defined as the largest width of the fascial as measured by the surgeon during the operation and was classified according to the European Hernia Society (EHS) classification system (W1<4 cm, W2=4-10 cm, W3≥10 cm). For mesh repairs, mesh position was intraperitoneal, preperitoneal or other (onlay/sublay/inlay). Mesh fixation techniques included tacking, suturing, or other (glue, clips, staples, or unspecified technique). In cases with tacked and sutured fixation, the technique was classified as tacking. The use of component separation technique during repair was also noted.

Outcomes

The primary outcome was recurrence after VHR. Recurrence was defined as the occurrence of a ventral hernia in the same scar as previous VHR noted by a physician. Recurrence was primarily determined by either physical examination or CT scans. The secondary outcomes were reoperation and postoperative surgical complications. The complications included were grades I-IV according to the Clavien-Dindo classification system. Complications including seroma, hematoma, wound infection, and abdominal wall abscess were defined as those occurring after the repair and associated with the surgical site. Only complications that were likely to be related to the previous VHR based on the medical records and operative notes were included. Reoperation was defined as any operation after the repair for complications such as seroma, hematoma, wound infection, mesh infection, or abdominal wall abscess.

Statistical analysis

Statistical analysis was performed using SPSS version 24 (IBM Corp, Armonk, NY). To analyze the independent risk factors after the VHR repairs, we performed bivariable analyses screening clinically relevant risk factors, including age, sex, use of mesh, mesh fixation technique, mesh position, component separation, hernia size, and preoperation comorbidities such as diabetes, hypertension, tobacco use, COPD, and obesity. We used a t-test for continuous variables, Pearson’s chi-square test for categorical variables, and Fisher's exact test for variables with an expected frequency less than 5. Univariate covariates were then entered simultaneously into a Cox regression model and multiple logistic regression model. Cox regression was used to evaluate the relationship between recurrence and clinical variables. The relationship between complications or reoperations and clinical variables was assessed using multivariate conditional regression analysis. All of the tests were two-tailed. P<0.05 was considered to be significant.

## Results

Between 2000 and 2009, 775 primary and incisional VHRs were performed at our institution. After excluding the repairs performed on patients with more than one VHR recurrence (129 cases) and infraumbilical/suprapubic repairs (148 cases), 420 patients were identified. During the follow-up period, 78 (18.57%) patients were lost to follow-up. One patient died on postoperative day 13 due to peritonitis leading to septic shock. Four patients had postoperative urinary retention. We analyzed 420 patients (median age 46 years [range 38-54]) including 230 females (54.8%). The procedures included were laparoscopic (n=31; 7.5%), laparoscopic converted to open (n=7; 1.7%), and open (n=372, 90%). Mesh was used in 74.2% of all repairs. The median patient follow-up was 137 months.

Characteristics of patients and repair

Patient demographics and repair characteristics are shown in Table 1. Prevalence of comorbidities like hypertension, diabetes, COPD, tobacco use, and obesity were similar in all four groups. Mesh use was more common in larger hernia repairs (p<0.0001) and was associated with longer operating times (p=0.10). Laparoscopic technique was the preferred technique in larger hernia repairs (p<0.0001) although it was also with longer operating times (p=0.01). Mesh position was intraperitoneal in most open repairs (47.5% vs 36.7% preperitoneal repairs). Mesh was tacked in most laparoscopic repairs but sutured in most open repairs. The length of postoperative hospital stay was similar in all groups.

**Table 1:** Patient demographics *Onlay, sublay, inlay, or plug +Other includes glue, clips, stymie pins, or not specified BMI, body mass index; COPD, chronic obstructive pulmonary disease; IQR, interquartile range; Lap, laparoscopic

Characteristic	Total (N=420)	Lap (N=31)	Open (N=389)	P Value	Mesh (N=312)	Suture (N=108)	P Value
Sex							
Female, n (%)	230 (54.8)	11 (35.5)	214 (56.3)	0.04	166 (53.7)	63 (58.3)	0.5
Male, n (%)	190 (45.2)	20 (64.5)	166 (43.7)		143 (46.3)	45 (41.7)	
Age, median (IQR) years	46 (38-54)	46 (33-51)	46 (38-54)	0.89	46 (38-55)	47 (39-53)	0.63
BMI, median (IQR)	30.9 (26.6-36.5)	31.7 (27.3-38.5)	30.8 (26.5-36.3)	0.97	30.8 (27.0-36.5)	31.2 (25.8-36.6)	0.86
Defect size, median (IQR) cm	6 (3-10)	10 (7-15)	6 (3-10)	<0.0001	8 (4-10)	4 (1-8)	<0.0001
Tobacco use, n (%)	159 (38.0)	7 (22.6)	152 (39.7)	0.08	118 (38.2)	41 (37.6)	0.91
Hypertension, n (%)	148 (35.7)	12 (38.7)	136 (35.8)	0.85	112 (36.6)	36 (33.0)	0.56
Diabetes, n (%)	72 (17.6)	4 (12.9)	68 (17.9)	0.63	59 (19.1)	15 (13.8)	0.24
COPD, n (%)	41 (9.8)	2 (6.5)	39 (10.3)	0.76	32 (10.4)	8 (7.3)	0.45
Previous abdominal surgery	371 (86.0)	30 (96.8)	335 (88.2)	0.23	285 (92.2)	86 (78.9)	<0.0001
Follow-up, median (IQR) months	137 (111-168)	157 (106-165)	136 (111-169)	0.56	137 (112-166)	134 (110-174)	0.89
Operating time, median (IQR) minutes	95 (50-155)	140 (95-205)	90 (50-100)	0.01	102 (60-160)	72 (35-130)	0.1
Postop stay, median (IQR) days	3 (1-5)	4 (1-5)	3 (1-5)	0.16	3 (1-5)	3 (0-6)	0.48
Mesh position, n (%)							
Intraperitoneal	158 (55.5)	31 (100)	132 (47.5)	-	-	-	-
Preperitoneal	105 (37.4)	NR	101 (36.7)		-	-	
Other*	20 (7.1)	NR	44 (15.8)		-	-	
Mesh fixation technique, n (%)							
Tacked	91 (29.2)	14 (45.2)	75 (27.5)	-	-	-	
Sutured	160 (51.3)	6 (19.4)	153 (56)		-	-	
Unspecified/other^+^	61 (19.6)	11 (35.5)	53 (16.5)		-	-	

Complications and reoperations

Among complications, wound infections were most common (16.2%), followed by seromas (11.3%) (Table 2). Mesh infection was a complication in 4.9% of all mesh repairs (n=312). The laparoscopic technique was associated with lower complications and reoperations rates as compared to the open technique. The overall complication rates for laparoscopic and open repairs were 25% and 26.8%, respectively, whereas the reoperation rates were 6.9% and 13.2%, respectively. However, upon multivariate analysis, no statistical difference was found in complication or reoperation rates between laparoscopic and open repairs (p=0.70 for complications and p=0.34 for reoperations).

**Table 2:** Ventral hernia repair outcomes *Adjusted p-values after logistic regression Lap, laparoscopic

Outcomes (%)	Total (N=420)	Lap (N=31)	Open (N=389)	P Value	Mesh (N=312)	Suture (N=108)	P Value
Any complication	26.6%	25.0%	26.8%	0.70*	25.7%	29.5%	0.10*
Wound infection (including mesh infection)	16.8%	7.1%	17.6%	0.19	14.9%	22.5%	0.09
Seroma	11.3%	21.4%	10.5%	0.17	12.0%	9.5%	0.24
Hematoma	3.9%	0.0%	4.3%	0.40	4.3%	2.9%	0.48
Abdominal wall abscess	10.3%	3.6%	11.1%	0.34	9.5%	12.7%	0.35
Reoperation	12.8%	6.9%	13.2%	0.34*	13.1%	10.8%	0.75*
Recurrence	13.6%	13.8%	13.6%	0.53*	12.8%	15.2%	0.67*

Logistic regression analysis for complications and reoperations is summarized in Table 3. VHRs with mesh had lower rates of complications (25.7% vs 29.5% for suture repairs) and slightly higher rates of reoperation (13.1% vs 10.8% for primary repairs). However, these differences were not significant after multivariate adjustment with logistic regression. After multivariate analysis, obesity, COPD, component separation technique, and prolonged operating time (>75th percentile) were associated with an increased risk of complications. Patients with BMI ≥ 30 had an odds ratio (OR) of 2.13 (95% CI 1.24 to 3.67; p=0.01) for developing complications like wound infection, seroma, hematoma, and abdominal wall abscess. Patients with COPD had an OR of 2.29 (95% CI 1.06 to 4.96; p=0.04) for developing complications. There were no significant interactions for analysis of complication rates. Use of component separation technique was associated with increased risk of complications (OR 5.67, 95% CI 1.04 to 31.08; p=0.05) and reoperations (OR 15.64, 95% CI 2.86 to 85.86; p<0.001). 

**Table 3:** Multivariate analyses of risk factors for complications and reoperations after ventral hernia repair using logistic regression BMI, body mass index; COPD, chronic obstructive pulmonary disease; Lap, laparoscopic

Variables	Complication		Reoperation	
	OR (95% CI)	P Value	OR (95% CI)	P Value
Sex (male)	1.51 (0.85-2.68)	0.16	1.61 (0.75-3.49)	0.22
Age (one year older)	1.00 (0.98-1.02)	0.98	1.00 (0.97-1.03)	0.87
BMI ≥ 30	2.13 (1.24-3.67)	0.01	1.48 (0.71-3.07)	0.30
Tobacco use	0.86 (0.51-1.45)	0.57	0.78 (0.38-1.58)	0.49
Hypertension	1.49 (0.87-2.54)	0.15	0.90 (0.42-1.95)	0.79
COPD	2.29 (1.06-4.96)	0.04	2.10 (0.78-5.64)	0.14
Previous abdominal surgery	2.31 (0.92-5.80)	0.08	0.91 (0.31-2.72)	0.87
Wound class (dirty)	0.86 (0.44-1.67)	0.66	1.07 (0.45-2.57)	0.88
Mesh use	0.62 (0.35-1.11)	0.10	1.13 (0.50-2.53)	0.77
Lap vs open	0.83 (0.32-2.18)	0.70	0.49 (0.10-2.28)	0.36
Component separation	5.67 (1.04-31.08)	0.05	15.27 (2.78-83.74)	<0.0001
Prolonged operating time	3.01 (1.71-5.31)	<0.0001	2.22 (1.04-4.75)	0.04
Defect size >4 cm	0.57 (0.3-1.07)	0.08	0.61 (0.27-1.41)	0.25

Recurrence rates

During the follow-up period, 13.6% of patients overall had at least one recurrence (Table 2). VHRs with mesh had lower rates of recurrence as compared to suture repairs (12.8% vs 15.2%, respectively; p=0.67); however, this difference was not statistically significant after Cox regression analysis. There was no significant difference in patients undergoing repair with laparoscopic or open technique (13.6% and 13.8%, respectively). The best-fitting Cox regression model included sex, age, obesity, tobacco use, hypertension, COPD, previous abdominal surgery, operative technique (laparoscopic vs open), mesh use, component separation technique, and prolonged operating time (Table 4). No significant risk factors for recurrence were identified after Cox regression analysis.

**Table 4:** Cox regression analysis of risk factors for recurrence BMI, body mass index; COPD, chronic obstructive pulmonary disease; Lap, laparoscopic

Variables	Recurrence	
	HR (95% CI)	P Value
Sex (male)	0.73 (0.390-1.34)	0.31
Age (one year older)	0.99 (0.97-1.02)	0.83
BMI ≥ 30	1.66 (0.92-2.99)	0.09
Tobacco use	1.36 (0.77-2.38)	0.29
Hypertension	1.03 (0.57-1.84)	0.93
COPD	1.65 (0.78-3.51)	0.19
Previous abdominal surgery	1.39 (0.54-3.72)	0.50
Mesh use	0.74 (0.40-1.35)	0.32
Lap vs open	1.12 (0.41-3.40)	0.75
Component separation	2.17 (0.51-9.82)	0.30

## Discussion

In our retrospective study of 420 patients who underwent primary or incisional VHRs between 2000 and 2009, we found that VHRs with laparoscopic technique and mesh use had lower rates of complications; however, this difference was not statistically significant after multivariate analysis. After a median follow-up time of 137 months, patients who underwent repair with mesh had a lower rate of recurrence (p=0.67). Furthermore, we identified obesity, COPD, prolonged operating time, and component separation technique as significant risk factors for complications, such as seroma, hematoma, wound infections, and abdominal wall abscess.

Our results, indicating the lack of differences in outcomes of laparoscopic and open repairs, are consistent with prior studies with similar long follow-up times, demonstrating that recurrence and complication rates did not differ between open and laparoscopic techniques [11-13]. A meta-analysis of randomized controlled trials showed that recurrence rates were similar for both techniques [13]. Another prospective study with long-term comparison of 710 repairs showed that quality of life (in short term), length of stay, and infection rates were decreased in laparoscopic repairs; however, overall long-term complications and recurrence rates were equal [14]. Previous studies have reported that mesh use, compared to suture repair, is associated with a lower risk of hernia recurrence and increased risk of infection [6]. Our study found lower rates of long-term recurrence and complications in patients who underwent VHR with mesh. Although these differences were not statistically significant, the differences in recurrence rates in our study are similar to those reported in literature. The lack of significance in our study can be attributed to low sample size.

Additionally, our analysis indicates higher rates of complications and reoperations in VHR with use of component separation technique. These results are likely attributable to the increased wound-healing complications and the already large defect size. A study of patients undergoing large VHRs showed that component separation technique may be ideal hernia repair for large defects; however, our results indicate significantly higher adverse outcomes of component separation technique in open VHRs [15,16].

Our findings are consistent with previous studies showing that obesity increases risk of complications after VHR [17,18]. A retrospective chart review study of 888 patients was analyzed to develop a risk-stratification scoring system for surgical site complications after VHR [18]. This study reported that a BMI≥40 had an OR of 3.2 for the development of surgical site infection, and mesh implant had an OR of 1.9 for the development of infection, seroma, hematoma, wound dehiscence, and/or fistula formation. Thus, there is a consensus on higher complication rates after VHR in morbidly obese patients, BMI≥40; however, our analysis indicates that obese patients (BMI≥30) undergoing VHR have an OR of 2.13 (95% CI 1.24 to 3.67, p=0.01) of postoperative complications [8,18]. The mechanisms underlying the increased complication rate in obese patients are likely multifactorial, but hypotheses exist that may explain the predisposition for complications in obese and hypertensive patients [10]. Impaired visualization due to body habitus in addition to defects in tissue structure and healing are likely contributors to postoperative complications after VHRs in obese patients [19].

Limitations of this study include small sample size, treatment facility bias, retrospective review, and significant loss of information due to data extraction from paper charts. Other data, such as the presence of multiple defects, location of hernia (for example, midline vs lateral), suture type, mesh types, preoperative medications, number of surgeons operating, and emergency vs elective procedures, were not available to be included in this study. Additionally, all patients had variable follow-up periods. In our Cox regression model, essential covariates like obesity lost significance because of our small sample size. Furthermore, mortality among the patients who were lost to follow-up was unknown. Therefore, it was not possible to report the overall mortality in this study.

Taken together, these findings suggest that variability in surgical outcomes exists for VHR in patients with obesity and preexisting hypertension. Addressing these modifiable risk factors in surgical candidates preoperatively may reduce the risk of complications and may guide the decision to undergo and timing of surgery.

## Conclusions

For patients undergoing VHR, obesity and COPD are significant risk factors for complications. Laparoscopic repairs had slightly lower rates of complication but similar rates of recurrence as open repairs after a long-term follow-up. Mesh repairs had lower rates of overall complications and recurrence. Mesh repairs should be considered in all patients, given that their complication rates were lower in our study, although not statistically significant. This study was limited by the number of patients with a complete follow-up available for analysis. Future studies looking into recurrence and complication rates in a larger, matched cohort may help confirm if long-term outcomes reported in this study are similar to those in the population. Obesity, COPD, and use of component separation technique are independent risk factors for complications after VHRs. Obesity is a modifiable risk factor, and future studies focused on these risk factors are necessary to revise treatment guidelines for VHR candidates with these comorbidities.
